# Editorial: Application in evolutionary novelties and diversities: Medicine, agriculture, and conservation

**DOI:** 10.3389/fgene.2022.1104836

**Published:** 2023-01-10

**Authors:** Jianhai Chen, Nikica Šprem, Yongjie Wu, Shengqian Xia

**Affiliations:** ^1^ Institutes for Systems Genetics, Frontiers Science Center for Disease-related Molecular Network, West China Hospital, Sichuan University, Chengdu, China; ^2^ Department of Fisheries, Apiculture, Wildlife Management and Special Zoology, Faculty of Agriculture, University of Zagreb, Zagreb, Croatia; ^3^ Key Laboratory of Bio-resources and Eco-environment of Ministry of Education, College of Life Sciences, Sichuan University, Chengdu, Sichuan, China; ^4^ Department of Ecology and Evolution, The University of Chicago, Chicago, IL, United States

**Keywords:** evolutionary young genes, genetic novelty, crop improvement, evolutionary biology, population genetics, biomarker, DNA sequencing

Since the early days of DNA sequencing using the Sanger dideoxy synthesis (Sanger et al., 1977) and Maxam–Gilbert chemical cleavage (Maxam and Gilbert, 1977) methods, multiple improved platforms have been developed and commercialized to produce higher throughput and longer DNA sequences. Example of these advances include the widespread exploration of high-throughput Next-Generation Sequencing (Slatko et al., 2018) (NGS) and long-reads oriented Third Generation Sequencing (Bleidorn, 2016) (TGS). The ever-growing amount of multi-omics data has subsequently innovated the research paradigm for biological questions, especially the population genetics which emphasized the classical evolutionary questions on genetic novelties and biodiversity.

As an old science, evolutionary biology occupies a central position in the biological sciences. Most evolutionary models and theories were developed early with the establishment of evolutionary biology and population genetics. Until recently, however, questions about the genetic basis of evolutionary novelty and biodiversity could be answered directly through in-depth analysis of sequences and molecular biology technologies. Our knowledge of biodiversity and novelty is growing rapidly and enormously in species of academic, social, and economic importance. In light of this critical bridging role of sequencing data and biotechnology between questions and applications, we have launched a Frontiers Research Topic: “*Application in Evolutionary Novelties and Diversities: Medicine, Agriculture, and Conservation, to yield more insightful intellectual chemicals*.”

Broadly speaking, the Research Topic aims to promote practical applications of genetic markers in medicine, agriculture, and conservation and exploration with evolutionary novelties and/or diversity. The novel genetic elements of a species have long attracted special interest. Among these markers, evolutionary young genes are known to play important roles in phenotypic novelties with medical importance (Suzuki et al., 2018), adaptive evolution (Deng et al., 2010; Long et al., 2013), agricultural potential applications (Xia et al., 2016; Chen et al., 2019; Zhang et al., 2020), genomic evolution of pest species (Miller et al., 2022), *etc.* In this Research Topic, young genes were identified and analyzed in a plant species, Olea europaea subsp. Cuspidate, a widespread close relative of olive. Notably, a burst (19.5%) of gene transposition events was detected in the common ancestor of olive subspecies, suggesting the importance of the emergence of new genes in the evolution of olive species.

In addition to evolutionarily young genes, other biomarkers, such as microsatellites, are also extensively explored for conservation genetics. In this Research Topic, the nuclear genetic diversity and structure of wild populations of Anastrepha ludens were analyzed with microsatellite markers. Gálvez-Reyes et al. examined nine microsatellite loci and answered interesting questions about population diversity, structure, gene flow, and population size of the Mexican fruit fly, *Anastrepha ludens*, an important pest that causes widespread damage to a range of fruit crops in Mexico. In addition, Sun et al. conducted a landscape genetic study and linked genetic differentiation to bioclimatic factors for the parasitoid *Stethynium empoasca*. Guan et al. produced a genome assembly for *Luehdorfia taibai,* an endangered butterfly endemic to the Qinling Moutains in China with extremely small populations, which showcases the whole-genome as a powerful and complete biomarker to study conservation biology. Ke et al. conducted temporal sampling and network analysis on *Plutella xylostella* and uncovered rapid population change and dynamic migration patterns in overwintering regions of this cosmopolitan pest. Guo et al. conducted a mendelian randomization study based on single-nucleotide polymorphisms (SNPs) to analyze the causal relationship between vitamin D and uterine fibroids, demonstrating the applications of genomic variants in addressing questions of cause and effect with medical considerations. Mamat et al. performed population genetics for *Tolai hares*, *Lepus tolai*, in Xinjiang, China, using genome-wide SNPs from SLAF-seq and mitochondrial markers. Mu et al. performed transcriptome analysis to understand the mechanisms underlying the adaptation of plateau pika, *Ochotona curzoniae*, on the Qinghai-Tibet Plateau. Tian and Ma proposed to use natural hybrids in horticulture to accelerate the breeding of woody ornamental plant. Although the specific questions are different, these studies demonstrate the power of genetic, genomic, or transcriptomic data at species or population level in address questions involving biodiversity and evolution.

In summary, this Research Topic has gained updated knowledge input from multidiscipline scientists who studied genetic markers considering the importance of novelty and diversity. Future fine-scale studies and multi-omics data from more diverse species and geographical populations would enrich our understanding of the evolutionary significance of genetic novelty and biodiversity on our planet.

## References

[B11] Bleidorn C. (2016). Third generation sequencing: technology and its potential impact on evolutionary biodiversity research. Syst. Biodivers. 14, 1–8.

[B1] Chen J. Mortola E. Du X. Zhao S. Liu X. (2019). Excess of retrogene traffic in pig X chromosome. Genetica 147, 23–32. 10.1007/s10709-018-0048-5 30535819

[B2] Deng C. Cheng C.-H. C. Ye H. He X. Chen L. (2010). Evolution of an antifreeze protein by neofunctionalization under escape from adaptive conflict. Proc. Natl. Acad. Sci. U. S. A. 107, 21593–21598. 10.1073/pnas.1007883107 21115821 PMC3003108

[B3] Long M. Vankuren N. W. Chen S. Vibranovski M. D. (2013). New gene evolution: Little did we know. Annu. Rev. Genet. 47, 307–333. 10.1146/annurev-genet-111212-133301 24050177 PMC4281893

[B4] Maxam A. M. Gilbert W. (1977). A new method for sequencing DNA. Proc. Natl. Acad. Sci. U. S. A. 74, 560–564. 10.1073/pnas.74.2.560 265521 PMC392330

[B5] Miller D. Chen J. Liang J. Betrán E. Long M. Sharakhov I. V. (2022). Retrogene duplication and expression patterns shaped by the evolution of sex chromosomes in malaria mosquitoes. Genes (Basel) 13, 968. 10.3390/genes13060968 35741730 PMC9222922

[B6] Sanger F. Nicklen S. Coulson A. R. (1977). DNA sequencing with chain-terminating inhibitors. Proc. Natl. Acad. Sci. U. S. A. 74, 5463–5467. 10.1073/pnas.74.12.5463 271968 PMC431765

[B7] Slatko B. E. Gardner A. F. Ausubel F. M. (2018). Overview of next-generation sequencing technologies. Curr. Protoc. Mol. Biol. 122, e59. 10.1002/cpmb.59 29851291 PMC6020069

[B8] Suzuki I. K. Gacquer D. Van Heurck R. Kumar D. Wojno M. Bilheu A. (2018). Human-specific NOTCH2NL genes expand cortical neurogenesis through delta/notch regulation. Cell 173, 1370–1384. e1316. 10.1016/j.cell.2018.03.067 29856955 PMC6092419

[B9] Xia S. Wang Z. Zhang H. Hu K. Zhang Z. Qin M. (2016). Altered transcription and neofunctionalization of duplicated genes rescue the harmful effects of a chimeric gene in Brassica napus. Plant Cell 28, 2060–2078. 10.1105/tpc.16.00281 27559024 PMC5059798

[B10] Zhang Z. Fan Y. Xiong J. Guo X. Hu K. Wang Z. (2020). Two young genes reshape a novel interaction network in Brassica napus. New Phytol. 225, 530–545. 10.1111/nph.16113 31407340

